# Multiple Modeling Techniques for Assessing Sesame Oil Extraction under Various Operating Conditions and Solvents

**DOI:** 10.3390/foods8040142

**Published:** 2019-04-25

**Authors:** Haitham Osman, Ihab Shigidi, Amir Arabi

**Affiliations:** 1 Department of Chemical Engineering, King Khalid University, P.O. Box 394, Abha 61411, Saudi Arabia; etaha@kku.edu.sa; 2 Department of Mechanical Engineering, King Khalid University, P.O. Box 394, Abha 61411, Saudi Arabia; aarabi@kku.edu.sa

**Keywords:** sesame oil extraction, response surface method, radial basis functions, artificial neural network

## Abstract

This paper compares four different modeling techniques: Response Surface Method (RSM), Linear Radial Basis Functions (LRBF), Quadratic Radial Basis Functions (QRBF), and Artificial Neural Network (ANN). The models were tested by monitoring their performance in predicting the optimum operating conditions for Sesame seed oil extraction yields. Experimental data using three different solvents—hexane, chloroform, and acetone—with varying ratios of solvents to seeds, all under different temperatures, rotational speeds, and mixing times, were modeled by the three proposed techniques. Efficiency for model predictions was examined by monitoring error value performance indicators (R^2^, R^2^_adj_, and RMSE). Results showed that the applied modeling techniques gave good agreements with experimental data regardless of the efficiency of the solvents in oil extraction. On the other hand, the ANN model consistently performed more accurate predictions with all tested solvents under all different operating conditions. This consistency is demonstrated by the higher values of R^2^ and R^2^_adj_ ratio equals to one and the very low value of error of RMSE (2.23 × 10^−3^ to 3.70 × 10^−7^), thus concluding that ANN possesses a universal ability to approximate nonlinear systems in comparison to other models.

## 1. Introduction

Sesame seed oil has many applications in health and food that have been known for several thousands of years. With higher oil content in comparison with other revivals, mechanical extraction for sesame seeds has always been the easiest in comparison to other seeds. Over the years the extraction process has undergone numerous developments and the principle of simply “squeeze the oil out” has been superseded significantly by the introduction of solvent extraction.

Sesame seeds have higher oil content (around 50%) than most of the known oilseeds. Sesame oil is known to be a high-priced and high-quality oil. It is also among the most stable edible oils despite its high degree of unsaturated fats [1,2]. Sesame oil is rich in monounsaturated and polyunsaturated fatty acids [3]. The most abundant fatty acids in sesame oil are oleic, linoleic, palmitic, and stearic acids, which together comprise about 96% of the total fatty acids. Oil content and fatty acid compositions vary significantly between oilseed crops and among the same crop collected from different geographical locations. Is has been reported that oil content for sesame seeds ranges between 44.6% to 53.1%. The content of oleic acid, linoleic acid, linolenic acid, palmitic acid, and stearic acid varied between 36.12–43.63%, 39.13–46.38%, 0.28–0.4%, 8.19–10.26%, and 4.63–6.35%, respectively [4].

Over the years, researchers investigated different solvents for sesame seed oil extraction [5] have investigated the impact of roasting seeds on the oil yield and have concluded that antioxidant capacity of the roasted seeds and oxidative stability of the extracted oil could be greater than that of the unroasted counterpart. The operating condition of sesame seed oil extraction has been studied in more comprehensive details by number of researchers [6], as they examined the effect of sesame seed particle sizes, the ratio of solvent to seed mass, contact time, stirring effect, roasting impact, and extraction temperatures.

The solvent extraction is the key point-operation. Extraction takes place due to the high affinity of solvents toward oil. The affinity is mainly chemically based. Many researchers have studied the impact of different solvents on sesame oil extraction, [7] using n-hexane, cyclohexane, and benzene, a mixture of n-hexane/chloroform (2:1, v/v), chloroform, acetic ether, butanol, and acetone; moreover, [8] they have used hexane and [9] used compressed propane and supercritical carbon dioxide. The majority of researchers have found that n-hexane yields higher extraction percentages, making it the optimum solvent. The use of clean technology for sesame oil extraction has been investigated by various researchers [10,11,12]. Different types of enzymes were used in aqueous solution in what is called Enzymatic-Assisted Aqueous Extraction (“EAAE”), i.e., (Perctinex Ultra SPL, Alcalase, alpha amylase, glucoamylase, pectinase, protease, lipase, and phytase) under different operating conditions. Results revealed that oil extraction quality was improved. However, oil yield was found to be lower in percentage in comparison to oil extracted using solvents and pressing [13,14].

The suitability of extraction methods, on the other hand, varies from plant to plant and there are significant differences in the capital and operation costs associated [15]. Different solvents have been studied by many researchers to reach higher extraction yields under economical operation conditions, i.e., temperature, mixing rate, and solvent-to-seed ratios, and reported results showed different effects on yield extraction by altering operating conditions [6,15,16].

Comparative studies between metamodels have been addressed by many researchers [17], comparing the predictions of ANN and RSM models of fatty acid methyl ester yield achieved from muskmelon oil under ultrasonication by two-step in situ process; other researchers applied RSM and ANN in modeling of extrusion process [18], modeling of microwave-assisted extraction methods [18,19], and modeling and optimisation of a heterogeneous photo-Fenton process [20].

In industry, the most advanced process control system requires accurate models if high performance is to be attained. Most chemical processes are nonlinear in nature, which makes developing precise models challenging [21,22].

When investigating the precision of the modeling technique, various factors, ranging from the nonlinearity of the model behaviour to the dimensionality and data sampling technique, to the internal parameters, are noticeably affected [23].

The need for a model that can accurately predict experimental behaviour has been the utmost challenge for researchers over the years; such models can dramatically reduce the time and operational cost in many engineering aspects. From here emerged the need to model sesame seed extraction using various solvents and under different operating conditions [21].

Some of the most recognized models that are used widely are the response surface models [23,24,25,26,27]. Extensive surveys and reviews of different meta-modeling methods and their applications are given in previous studies [28,29,30]. On the other hand [31], RSM and RBF were studied to find the best method for modeling highly nonlinear responses found in impact-related problems. They also compared the RSM and RBF models with a highly nonlinear test function. Despite the computation cost of RBF, they concluded supremacy of RBF over RSM in such optimization problems.

## 2. Methodology

The previously obtained experimental data for sesame oil extraction [6] were modeled using different solvents, namely hexane, acetone, and chloroform. These solvents were investigated as they report to have higher extraction yields. Experimental data were obtained for different sesame seeds average particle sizes (2, 1.5, 1, 0.8, and 0.5 mm) after roasting at different temperatures (100, 120, 140, 160, 180, and 200 °C) as a pre-treatment process. Different ratios of sesame seed mass to solvent mass (1:1, 1:2, 1:3, 1:4, and 1:5) and contact time of 6, 12, and 24 hours with varying stirring speeds of 0, 150, 300, and 700 rpm were examined and samples were subjected to heating at different temperatures (25, 30, 35, 40, 45, and 50 °C) during contact period of extraction; data obtained at 40 °C were used in this work as it gave maximum extraction yield [6]. Extracted oil was then separated by distillation. Oil yield was calculated as a ratio of extracted oil to seed weight. Experimental results used for modeling are presented in Appendix A.

### 2.1. Modeling Techniques

In this paper the following models are used: Response Surface Method, Linear Radial Basic Function, Quadratic Radial Basic Function, and Artificial Neural Network. The four promising modeling techniques, LRBF, QRBF, ANN, and RSM, were applied to model the experimentally available data, from which the predictions generated for oil extraction yields were obtained and then compared to evaluate these models’ adeptness.

#### 2.1.1. Response Surface Methodology (RSM)

Response surface methodology came from the original work of a previous study [24]. Their collaboration was initiated at a chemical company when solving the problem of determining optimal operating conditions for chemical processes. Response surface methodology is used in many practical applications in which the goal is to identify the levels of design factors or variables that optimize a response. Despite its simplicity and efficiency, RSM provides efficient and accurate solutions. Therefore, it has successfully been applied in many engineering problems [32,33,34,35].

RSM is a higher order polynomial model; a second-order (Quadratic) polynomial equation is developed after ANOVA test to express the value of the variable *Y* (oil Yield) as a function of each independent variable (X_1_, X_2_, and X_3_), as follows [16]:

(1) $$Y=\beta_{o}+\sum_{i=1}^{3}\beta_{i}X_{i}\;+\sum_{i=1}^{3}\beta_{ii}X_{i}^{2}+\sum \sum_{i<j=1}^{3}\beta_{ii}X_{i}X_{j},$$

where *β*_0_, *β_i_*, *β_ii_*, and *β* in are the regression coefficients for intercept, and the notations X_1_ = A, X_2_ = B, and X_3_ = C are the independent variables, as presented in Table 1. A least-squares methods can be used to determine the parameters for RSM as follows:

*β* = (**X**^T^**X**)^−1^**X**^T^**Y**, (2)

All regression models were developed using the Design of Experiment, DOE and statistical toolbox in MATLAB^TM^.

#### 2.1.2. Linear and Multiquadric Radial Basis Function (LRBF and QRBF)

A Radial Basis Function (RBF) is a real-valued function that depends only on the distance from the origin, Any function ϕ that satisfies the property ϕ (x) = ϕ (ǁ x ǁ) is a radial function. Even though the norm is usually Euclidean distance, other distance functions can also be possible [36]. RBF uses a series of basic functions that are symmetric and cantered at each sampling point, and it was originally developed for scattered multivariate data interpolation [25]. RBF had applications in medical imaging, ocean depth measurement, altitude measurement, rainfall interpolation, surveying, mapping, geography and geology, and image warping [37].

If f(x) is the true objective or response function and *f*’(*x*) its approximation obtained from a classical RBF with the general form:

(3) $$f\prime \left(x\right)=\sum_{i=1}^{n}\lambda_{i}\varphi \left(\left\|x-x_{i}\right\|\right),$$

where *n* is the number of sampling points, x is the vector of design variables, *x_i_* is the vector of design variables at the *i*-th sampling point, $\left\|x-x_{i}\right\|$ is the Euclidean distance, *φ* is a basis function, and *λ_i_* is the unknown weighting coefficient.

Therefore, an RBF is actually a linear combination of *n* basis functions with weighted coefficients. Some of the most commonly used basis functions include:

- Linear Radial Basis Function (LRBF): *φ(r)* = *r*.
- Gaussian: $\varphi \left(r\right)=e^{-cr^{2}},\;0<c\leq 1$.
- Quadric Radial Basis Function (QRBF): $\varphi \left(r\right)=\sqrt{r^{2}+c^{2}},\;0<c\leq 1$.
- Inverse multiquadric: $\varphi \left(r\right)=\frac{1}{\left(r^{2}+c^{2}\;\right)},\;0<c\leq 1$.

An RBF using the highly nonlinear functions does not work well for linear responses [38]. To solve this problem, an augmented RBF polynomial function is included:

(4) $$f\prime \left(x\right)=\sum_{i=1}^{n}\lambda_{i}\varphi \left(\left\|x-x_{i}\right\|\right)+\sum_{j=1}^{n}c_{j}p_{j},$$

where *n* is the total number of terms in the polynomial, and *c_j_* (*j* = 1,2,…, *m*) is the corresponding coefficient. A detailed discussion on the polynomial functions that may be used can be found in a previous study [38].

RBF passes through all the sampling points exactly. This means that function values from the approximate function are equal to the true function values at the sampling points. Therefore, it would not be possible to check RBF model fitness with ANOVA, which is the main drawback of RBF.

All RBF have been claimed to create better models than the RSM with a limited number of samples [31]; it has not been found from the literature which RBF or RBFs are highly accurate in general for linear, quadratic, and high-order nonlinear responses. A study on the accuracy of RBF models is needed before RBF can be used to create high-fidelity global models because the types of responses are typically unknown in most situations.

#### 2.1.3. Artificial Neural Network (ANN)

ANN is made up of two parts, nodes and connections. Nodes consist of neurons, which consist of the transfer function that takes the argument S, and produces the scalar output of a single neuron. The most used transfer functions to solve linear and nonlinear regression problems are purelin, logsig, and tansig [39].

For the case of logistic output the log sig transfer function may be written as:

(5) $$logsig\left(S\right)=\frac{1}{1+e^{\left(-S\right)}},$$

**Figure 1:**
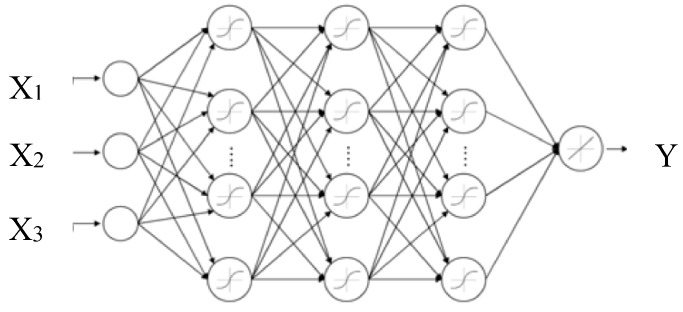
Architecture of the ANN with Multi-Layer Perceptron (MLP), with three inputs and one output.

The architecture of the neural network is presented in the form in which the neurons’ inputs and outputs are connected. These neurons are divided into several groups, called layers. A multi-layer neural network has hidden and output layers consisting of hidden and output neurons, respectively. Frequently, the inputs are considered as an additional layer. The most common neural network architecture used for solving nonlinear regression problems is the multi-layer feed-forward neural network, also known as Multi-Layer Perceptron (MLP), as shown in Figure 1.

A technique called “Early Stopping” was used during model training to avoid overfitting and subsequent poor generalization. Data sets were divided into 70% training set, 20% testing set, and 10% validation set. The number of training samples was 42, number of testing samples was 12, and validation was 6 samples. The MATLAB Neural Network Toolbox, version 6, was used to design and implemented all the ANNs.

### 2.2. Model Validation and Evaluation

In order to evaluate the goodness of the model fitting and prediction accuracy of the constructed models, *R*^2^ and error analyses were performed between the experimental and predicted data in the LRBF, QRBF, RSM, and ANN models. Many approaches for validation stated in the literature are used for error analyses, with some listed in a previous study [36].

In this paper, promising techniques that used the error as a performance index to measure the model accuracy are introduced. There are a number of different measures of model accuracy. The first two are the root mean square error (RMSE) and the R square value, are defined below:

(6) $$\mathrm{RMSE}=\sqrt{\frac{{\sum_{i=1}^{m}\left(y-\hat{y}\right)}^{2}}{m}},$$

(7) $$R^{2}=\frac{\sum_{i=1}^{m}{\left(y-\hat{y}\right)}^{2}}{\sum_{i=1}^{m}{\left(y-\bar{y}\right)}^{2}},$$

where $\hat{y}$ is the predicted value, *y* is the mean of the observed values.

In general, the larger the values of *R*^2^ and *R*^2^_*adj*_, and the smaller the value of RMSE, the better the fit. In situations where the number of design variables is large, it is more appropriate to look at *R*^2^_*adj*_, because *R*^2^ always increases as the number of terms in the model is increased, while *R*^2^_*adj*_ actually decreases if unnecessary terms are added to the model [19]. The four techniques proposed in this study are used to examine experimental data for solvent extraction of sesame seeds using three solvents, chloroform, acetone, and hexane. The experiments were conducted under different operating conditions (temperature, mixing speed, and solvent/seed ratio); experimental results are presented in a previous work [6,16]. Different statistical analysis techniques, e.g., ANOVA test, can be used to check the fitness of an RSM model, and hence identify the main effects of design variables. However, the main effect analysis is not the focus of this study and will not be discussed here. The major statistical parameters used for evaluating model fitness are the *R*, adjusted *R*^2^, and Root Mean Square Error (RMSE). Note that, these parameters are not totally independent of each other and are calculated by the methods listed in the following section.

#### 2.2.1. Root Mean Square Error (RMSE)

Generally speaking, the smaller the value of RMSE, the better the fit. It can be calculated as:

(8) $$\mathrm{RMSE}=\sqrt{\frac{SSE}{n-p-1}},$$

where *p* is the number of non-constant terms in the RSM model, SSE is the sum of square errors, and SST is the total sum of squares. SSE and SST are calculated as:

(9) $$\mathrm{SSE}=\sum_{i=1}^{n}\left(fi-f^{\prime }\right),$$

(10) $$\mathrm{SST}=\sum_{i=1}^{n}\left(fi-\bar{f}\right),$$

where *fi* is the measured function value at the *i*-th design point, *fi* is the function value calculated from the polynomial at the *i*-th design point, and *f* is the mean value of *fi*.

#### 2.2.2. *R*^2^ and *R*^2^*_adj_*

In situations where the number of design variables is large, it is more appropriate to look at *R*^2^*_adj_*, because *R*^2^ always increases as the number of terms in the model is increased:

(11) $$R^{2}=1-\frac{\mathrm{SSE}}{SST},$$

*R*^2^_*adj*_ actually decreases if unnecessary terms are added to the model,

(12) $$R_{adj}^{2}=1-\left(1-R\right)\frac{n-1}{n-p-1}.$$

## 3. Results and Discussion

The model prediction is developed using MATLAB 2017a with Model-Based Calibration Toolbox™ in windows 7 platform with i5 8GB RAM. This toolbox uses Design of Experiments (DoE), statistical modeling, and optimization techniques to efficiently produce high quality calibrations for the oil yield model. To evaluate the computational efficiency and accuracy of the developed models, the above performance evaluation functions are known as good indicators. The small values of *R*^2^_*adj*_ and *R*^2^, as well as large values of RMSE, indicate bad fittings for the RSM models. Using the same experimental data samples, RBF models with the linear function (LRBF) and multi-quadric functions (QRBF) and the ANN model are also developed.

### 3.1. Modeling Experimental Data Using RSM

**Figure 2:**
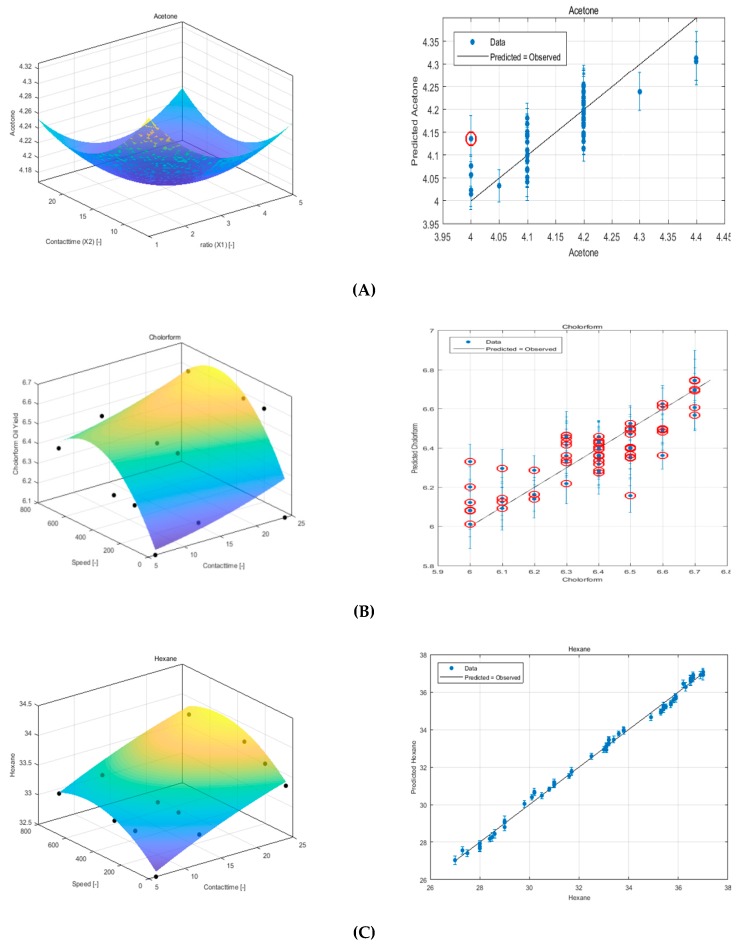
Response surface plot of using RSM and experiment versus predicted data for Sesame seeds oil extraction using (**A**) acetone, (**B**) chloroform, and (**C**) hexane.

A second-order (Quadratic) polynomial optimum equation was developed after testing the feasibility of other possible orders (2–6 orders, see Figure 2). To express the value of the variable Y (oil Yield) as a function of each independent variable (*X*_1_, *X*_2_ and *X*_3_), the following models are obtained.

#### 3.1.1. Acetone

The second order equation for acetone is:

$$Y\left(\mathrm{oil}\;\mathrm{Yield}\right)=4.17-0.024\times X_{2}+0.0641\;\times X_{3}+0.0183\times X_{1}-0.047\times X_{2}{}^{2}-0.0119\times X_{2}\times X_{3}-0.012\times X_{2}\times X_{1}-0.096\times X_{3}{}^{2}-0.0122\times X_{3}\times X_{1}-0.0511\times X_{1}{}^{2}$$

#### 3.1.2. Chloroform

The second order equation for Chloroform is:

$$Y\left(\mathrm{oil}\;\mathrm{Yield}\right)=6.545+0.088\times X_{2}+0.15\times X_{3}-0.068\times X_{1}+0.0206\times X_{2}{}^{2}+0.0028\times X_{2}\times X_{3}-0.10812\times X_{2}\times X_{1}-0.2003\times X_{3}{}^{2}-0.01759\times X_{3}\times X_{1}-0.05714\times X_{1}{}^{2}$$

#### 3.1.3. Hexane

$$Y\left(\mathrm{oil}\;\mathrm{Yield}\right)=33.6575+0.427709\times X_{2}+0.252519\times X_{3}+4.25703\times X_{1}-0.06\times X_{2}{}^{2}-0.0128276\times X_{2}\times X_{3}-0.259821\times X_{2}\times X_{1}-0.320569\times X_{3}{}^{2}-0.0976245\times X_{3}\times X_{1}-0.928571\times X_{1}{}^{2}$$

### 3.2. Modeling Experimental Data Using LRBF

**Figure 3:**
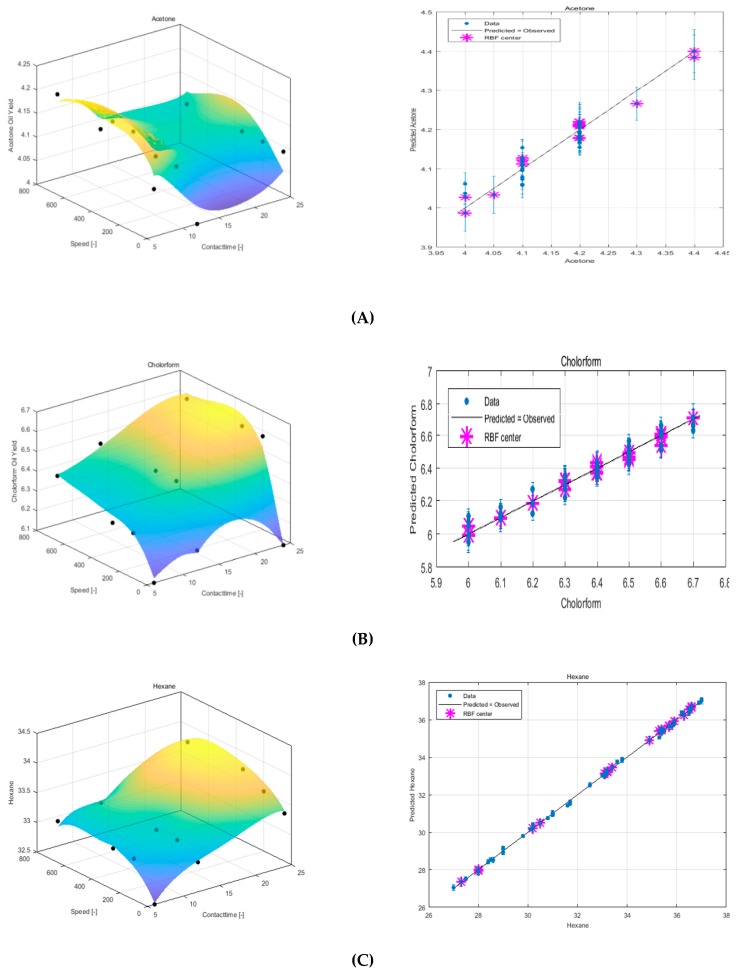
Response surface plot of using LRBF and experimental versus predicted data for sesame seed oil extraction using (**A**) acetone, (**B**) chloroform, and (**C**) hexane.

The response surface model for LRBF for the three solvents is shown in Figure 3. In the graph the predicted oil extraction yields versus experimental data are presented. The result showed better agreement between predicted and experimental data. When comparing these results with that obtained using the RSM model, the RBF model prevails. Moreover, hexane showed the best RBF linear model agreement between the predictions versus experimental data of the oil yield extracted in comparison to other solvents. The RBF linear model showing higher *R*^2^ and *R*^2^*_adj_* values at near to 1 (Table 4), whereas for the RMSE value, hexane has been found to achieve the highest value of 0.116 in comparison to the other solvents. Table 2 shows the linear RBF optimum parameters.

### 3.3. Modeling Experimental Data Using QRBF

**Figure 4:**
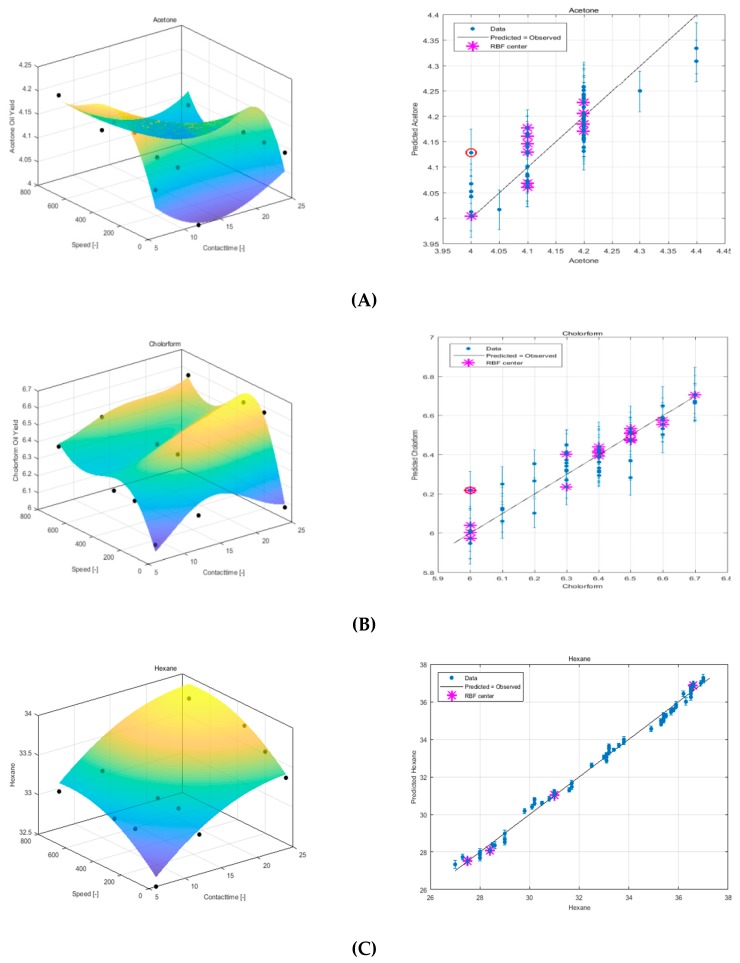
Response surface plot using QRBF and experimental versus predicted data for sesame seed oil extraction using (**A**) acetone, (**B**) chloroform, and (**C**) hexane.

Figure 4 shows the difference between the three solvent models. It can be seen from the graph of the experiment versus predicted oil yield data that the hexane solvent produced a more robust model when compared to the other solvents. The QRBF parameter values can be seen in Table 2.

### 3.4. Modeling Experimental Data Using ANN

**Figure 5:**
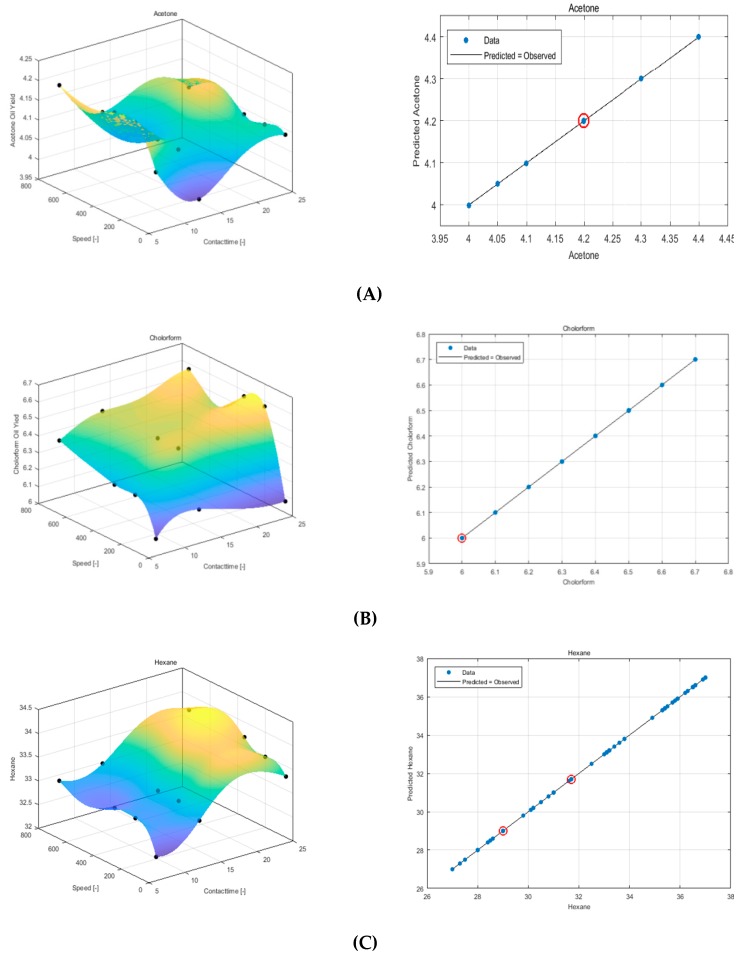
Response surface plot using ANN and experimental versus predicted data for sesame seed oil extraction using (**A**) acetone, (**B**) chloroform, and (**C**) hexane.

The optimum configuration for the neural network is performed with 2 hidden layers; the first layer contains 10 neurons, the second layer 5 neurons. Different back-propagation (BP) algorithms were compared to select the best-suited BP algorithm. The Marquardt–Levenberg learning algorithm was used with a Mean Squared Error (MSE). Table 3 shows the ANN optimum parameter values used for the three solvents. The ANN model and its parameters variation were determined based on the minimum values of MSE of the training dataset. The 3D response surface plot using ANN for all solvents together with a graph presenting experimental versus predicted oil yield data is shown in Figure 5. The result shows identical matching between experimental and predicted data, thus ANN overperformed all the aforementioned models in term of low RSME, (acetone = 3.7 × 10^−5^, chloroform = 3.3757 × 10^−5^, and hexane = 2.23 × 10^−3^) and *R*^2^ and *R*^2^_*adj*_ equal one.

The results summarized in Table 4 show that values of *R*^2^, *R*^2^_*adj*_, for the RSM Quadratic model indicate good agreement for the hexane solvent with *R*^2^, *R*^2^_*adj*_ near to one, whereas, the RMSE value for the hexane was relatively large (0.225) compared to the rest of models. On the other hand, ANN has a smaller value of RMSE and *R*^2^, *R*^2^_*adj*_ equal to one, indicating the most accurate modeling for all three solvents.

The LRBF gave better *R*^2^, *R*^2^_*adj*_ values in comparison with the RSM quadratic for all three solvents and showed better responses for Chloroform. The QRBF showed values for *R*^2^, *R*^2^_*adj*_ equal to one for all modeled solvents.

In a nutshell, the results show the supremacy of the ANN over the other modeling techniques applied in terms of minimum RMSE, and *R*^2^, *R*^2^_*adj*_ values near one. This result agrees with that obtained by many researchers confirming that the ANN model has the best prediction [17,18,20,36].

## 4. Conclusions and Future Work

The systematic comparative study presented in this paper has provided insightful observations into the performance of various meta-modeling techniques. This study has revealed that the properly trained ANN model has consistently performed more accurate prediction compared to those of RSM, Linear (LRBF), and Multi-quadric (QRBF) models in all aspects. This accurateness is expressed in the very high values of *R*^2^ and *R*^2^*_adj_* ratios equal to one and the very low value of error for RMSE (for hexane 2.23 × 10^−3^, chloroform 3.3757 × 10^−5^, and for acetone 3.7 × 10^−5^) indicators for the ANN results compared to others. This confirms that the ANN model displays a significantly higher generalization capacity than the rest of the models. The reason can be accredited to the universal ability of ANN to approximate the nonlinearity of the system.

As a conclusion it can be noted from the plot of experimental data against the predicted data that the ANN is superior, and the modeling techniques compared to RSM, Linear (LRBF), and quadric (QRBF) in the second-ranking QRBF proved to be more accurate and had the finest prediction capability, when compared to LRBF and RSM. The applications of artificial neural networks can be used for on-line state estimation and control of sesame oil extraction.

Statistical indices have generated competitive results in predicting experimental extraction data. It is recommended that these techniques be applied on further techniques, such as modeling green solvent systems. Moreover, the experimental testing of different solvent mixtures in addition to analysing extracted oil quality by monitoring different properties, such as pH, acidity, and peroxide value, can be introduced as extra operating condition functions to be modeled.

## Figures and Tables

**Table 1:** Experimental design variables used for modeling Sesame seeds oil yield.

Independent Variables *X*_n_		Coded Levels		
		−1	0	1
*X*_1_: Seeds to solvent ratio (%)	A	1	3	5
*X*_2_: Contact Time (hr)	B	6	12	24
*X*_3_: Stirring speed (rpm)	C	0	350	700

**Table 2:** QRBF parameters Value.

Solvents	Model	Centers	Width	Regularization Parameter λ
Acetone	Linear RBF	20	0.0593	0.0487
	Multi-quadric RBF	11	2.718	2.471
Chloroform	Linear RBF	20	0.055952	0.03144
	Multi-quadric RBF	19	1.432	2.13 × 10^−5^
Hexane	Linear RBF	20	0.0683	0.03375
	Multi-quadric RBF	4	4.75	4.399 × 10^−5^

**Table 3:** ANN parameters value.

Parameter	Value
Number of input variables	3
Number of first layer neurons	10
Number of output neurons	5
Learning rule	Levenberg–Marquardt
Number of iteration	1000
Error goal	0.0001
Mu	0.0005

**Table 4:** Model Performance validation.

Solvents	Model Name	Observations	RMSE	*R* ^2^	*R* ^2^_*adj*_
**Hexane**	RSM Quadratic	60	0.225	0.996	0.995
	ANN [5,10]	60	2.23 × 10^−3^	1	1
	LRBF	60	0.116	0.998	0.999
	QRBF	60	0.263	1	1
**Chloroform**	RSM Quadratic	60	0.123	0.693	0.638
	ANN [5,10]	60	3.3757 × 10^−5^	1	1
	LRBF	60	0.053	0.912	0.933
	QRBF	60	0.086	1	1
**Acetone**	RSM Quadratic	60	0.05	0.692	0.636
	ANN [5,10]	60	3.7 × 10^−5^	1	1
	LRBF	60	0.03	0.852	0.871
	QRBF	60	0.047	1	1

## Appendix A

**Seeds/Solvent** **Ratio**	**Contact Time** **(hour)**	**Stirring Speed** **(rpm)**	**Oil Yield %**		
			**By Chloroform**	**By Acetone**	**By Hexane**
1:1	6	0	6	4	27
1:1	6	150	6.3	4.3	27.5
1:1	6	300	6.5	4.4	28
1:1	6	700	6.5	4.4	28
1:1	12	0	6	4	27.3
1:1	12	150	6	4.2	28
1:1	12	300	6.3	4.2	28.4
1:1	12	700	6.3	4.2	28.5
1:1	24	0	6.5	4.1	28.6
1:1	24	150	6.7	4.2	29
1:1	24	300	6.7	4.2	29
1:1	24	700	6.7	4.2	29
1:2	6	0	6	4.1	29.8
1:2	6	150	6.2	4.2	30.1
1:2	6	300	6.3	4.2	30.2
1:2	6	700	6.5	4.2	30.2
1:2	12	0	6.5	4.05	30.5
1:2	12	150	6.6	4.2	30.8
1:2	12	300	6.6	4.2	31
1:2	12	700	6.6	4.2	31
1:2	24	0	6.3	4.1	31
1:2	24	150	6.7	4.2	31.6
1:2	24	300	6.7	4.2	31.7
1:2	24	700	6.7	4.2	31.7
1:3	6	0	6.1	4.1	32.5
1:3	6	150	6.3	4.2	33.1
1:3	6	300	6.3	4.2	33.1
1:3	6	700	6.4	4.2	33.1
1:3	12	0	6.2	4	33
1:3	12	150	6.5	4.1	33.2
1:3	12	300	6.5	4.1	33.2
1:3	12	700	6.5	4.1	33.2
1:3	24	0	6.1	4.1	33.4
1:3	24	150	6.6	4.1	33.6
1:3	24	300	6.6	4.1	33.8
1:3	24	700	6.6	4.1	33.8
1:4	6	0	6.2	4.1	34.9
1:4	6	150	6.4	4.2	35.3
1:4	6	300	6.4	4.2	35.4
1:4	6	700	6.4	4.2	35.4
1:4	12	0	6.1	4	35.3
1:4	12	150	6.3	4.2	35.5
1:4	12	300	6.3	4.2	35.7
1:4	12	700	6.3	4.2	35.7
1:4	24	0	6	4	35.4
1:4	24	150	6.5	4.1	35.8
1:4	24	300	6.5	4.2	35.9
1:4	24	700	6.5	4.2	35.9
1:5	6	0	6.1	4.1	36.3
1:5	6	150	6.4	4.2	36.5
1:5	6	300	6.4	4.2	36.5
1:5	6	700	6.4	4.2	36.5
1:5	12	0	6.1	4	36.2
1:5	12	150	6.4	4.2	36.5
1:5	12	300	6.4	4.2	36.6
1:5	12	700	6.4	4.2	36.6
1:5	24	0	6	4.1	36.5
1:5	24	150	6.4	4.2	36.9
1:5	24	300	6.4	4.2	37
1:5	24	700	6.4	4.2	37
